# *Fluocell* for Ratiometric and High-Throughput Live-Cell Image Visualization and Quantitation

**DOI:** 10.3389/fphy.2019.00154

**Published:** 2019-10-23

**Authors:** Qin Qin, Shannon Laub, Yiwen Shi, Mingxing Ouyang, Qin Peng, Jin Zhang, Yingxiao Wang, Shaoying Lu

**Affiliations:** 1 Department of Bioengineering, Institute of Engineering in Medicine, University of California, San Diego, San Diego, CA, United States; 2 Department of Mathematics, Center of Computational Mathematics, University of California, San Diego, San Diego, CA, United State; 3 Institute of Biomedical Engineering and Health Sciences, Changzhou University, Changzhou, China; 4 Department of Pharmacology, University of California, San Diego, San Diego, CA, United States

**Keywords:** ratiometric, high-throughput, live-cell image, visualization, quantitation, image analysis

## Abstract

Spatiotemporal regulation of molecular activities dictates cellular function and fate. Investigation of dynamic molecular activities in live cells often requires the visualization and quantitation of fluorescent ratio image sequences with subcellular resolution and in high throughput. Hence, there is a great need for convenient software tools specifically designed with these capabilities. Here we describe a well-characterized open-source software package, *Fluocell*, customized to visualize pixelwise ratiometric images and calculate ratio time courses with subcellular resolution and in high throughput. *Fluocell* also provides group statistics and kinetic analysis functions for the quantified time courses, as well as 3D structure and function visualization for ratio images. The application of *Fluocell* is demonstrated by the ratiometric analysis of intensity images for several single-chain Förster (or fluorescence) resonance energy transfer (FRET)-based biosensors, allowing efficient quantification of dynamic molecular activities in a heterogeneous population of single live cells. Our analysis revealed distinct activation kinetics of Fyn kinase in the cytosolic and membrane compartments, and visualized a 4D spatiotemporal distribution of epigenetic signals in mitotic cells. Therefore, *Fluocell* provides an integrated environment for ratiometric live-cell image visualization and analysis, which generates high-quality single-cell dynamic data and allows the quantitative machine-learning of biophysical and biochemical computational models for molecular regulations in cells and tissues.

## INTRODUCTION

The localization and activity of intracellular molecules have been successfully monitored with chimeric fluorescence proteins at single-cell levels to reveal how they dictate cellular function and fate [1–3]. However, intensity-based measurements can be artificially affected by different reporter expression levels in individual cells and subcellular variation of protein distribution due to cellular compartments and membrane folding [2, 3]. Therefore, ratiometric visualization and analysis are often necessary to normalize the fluorescence signals by a reference in the same cell, and to render the results independent of artifacts [3, 4]. Furthermore, intrinsically ratiometric single-chain FRET biosensors have been widely applied to monitor subcellular dynamic molecular activities with high spatiotemporal resolution [5, 6], which also require ratiometric analysis. The single-chain FRET biosensors ensure 1:1 donor-acceptor ratio and allow the quantification of FRET signal without cross-talk. Currently, intensity-based software packages have been developed with enriched functionalities with graphic user interfaces [7–10], while some general open-source ratiometric image analysis tools can be used for time-course quantifications with programming and customization [11, 12]. However, existing ratiometric analysis tools lack desired flexibility in preprocessing and quantification options and have not been widely used [13, 14].

At this front, we developed the *Fluocell* software package to visualize and quantify dynamic sequences of ratiometric image data with subcellular resolutions and in high throughput. *Fluocell* has been developed over many years with its designed functions extensively tested and refined. It is also accompanied with a detailed documentation. *Fluocell* is built with a graphic user interface (GUI) in the Java programming language and functions in MATLAB to visualize and quantify pixelwise intensity ratio images. The extension module, *Quanty*, is developed on top of *Fluocell* to allow automatic and high-throughput ratiometric processing. *Fluocell* and *Quanty* are cross-platform compatible, object-oriented, and modularized. The source code, documentation and example data are freely available at our group website and GitHub. The ratiometric image analysis functionalities of *Fluocell* have been extensively tested by us and other groups [15–21]. In addition, *Fluocell* also contains a previously published *Diffusion* module for image-based FRAP analysis, as well as intensity-based polarity analysis functions which have been used to quantify the spatial distribution of PI3K and Rac1 in polarized cells seeded on micropatterns [22–24].

The application of *Fluocell* and *Quanty* is demonstrated by the pixelwise ratiometric analysis of intensity images of several FRET-based biosensors. Single-chain protein tyrosine kinase biosensors have been widely applied to visualize the spatiotemporal distribution of kinase activity in live cells [3, 6, 23, 25, 26]. These biosensors contain an enhanced cyan fluorescent protein (ECFP as the FRET donor), a Src SH2 domain, a flexible linker, a specific tyrosine-containing substrate peptide, and a yellow fluorescent protein (YPet as the FRET acceptor, Figure S1) [23, 26, 27]. Active kinases can promote tyrosine phosphorylation on the substrate peptide of the corresponding biosensors, leading to a subsequent conformational change, and a decrease of FRET efficiency between the donor and the acceptor, while phosphatase works reversely to dephosphorylate the peptide and cause increase of FRET (Figure S1). Therefore, the donor/acceptor emission ratio of the biosensor signals represent local biosensor phosphorylation mediated by the specific kinase in live cells. These biosensors can also be genetically engineered to localize at different subcellular compartments, including the plasma membrane micro-domains, and to provide versatile measurement of local molecular activities [6, 28]. As such, the ratiometric and high-throughput visualization and quantification of signals from these FRET biosensors can provide important information of spatiotemporal enzymatic activity at the single-cell level in a heterogeneous cell population.

With the functionalities of *Fluocell* and *Quanty*, gigabytes of dynamic image data can be viewed and quantified in an automated workflow, within a matter of minutes. The software packages enable the efficient investigation of molecular activation kinetics in a large population of single cells. In our study, the quantified results from *Fluocell* and *Quanty* revealed different activation kinetics of Fyn kinase in the cytosolic and membrane compartments, and visualized a 4D distribution of epigenetic histone methylation signal in mitotic cells. Although FRET ratiometric imaging were used as an example, the functionality of *Fluocell* and *Quanty* is general and can be applied to any pixelwise ratiometric analysis. Overall, *Fluocell/Quanty* provides an integrative environment for live-cell ratiometric image visualization and analysis, which will ultimately generate large amount of high-quality molecular data and allow the machine-learning of a comprehensive molecular regulation map for all cell types. In this paper, we describe the systematic design, functional characterization, and application with specific biological problems.

## RESULTS

### The Ratiometric Image Analysis Workflow of *Fluocell* and *Quanty*

**FIGURE 1 |:**
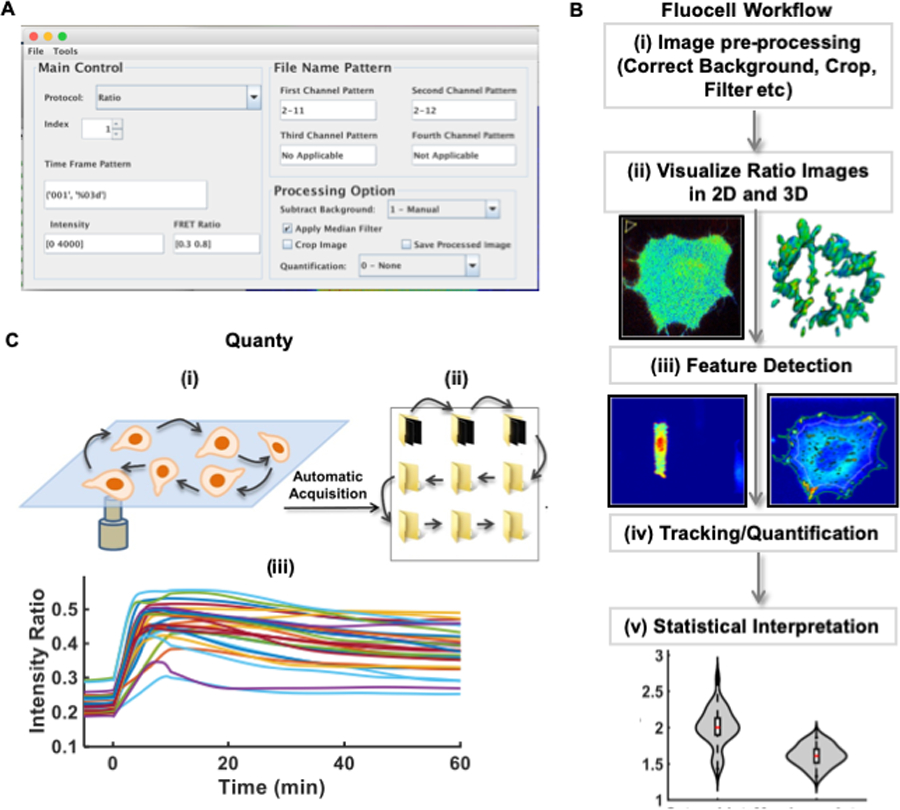
*Fluocell* overview and features. **(A)** A screenshot of the main *Fluocell* interface; **(B)** Schematic of a typical ratio image visualization and analysis pipeline of *Fluocell*; **(C)** The multiple-position quantification feature in the *Quanty* module: (i) The video image data of multiple cells are obtained by a microscope with an automated stage. (ii) The image files for each cell are sorted into a folder by the *batch_sort_file_multiple_position* function; the *g2p_quantify* function automatically scans the list of folders and quantifies the time courses of intensity ratio for all cells. (iii) The time courses are collected and plotted by the *group_plot* function to allow further analysis.

Ratiometric image analysis is widely used since the self-normalization process permits analysis of molecular concentration or activity independent of variable protein expression levels among different cells or different subcellular regions. The *Fluocell* image analysis software package is specifically designed for the ratiometric quantification of live-cell imaging data such as those recorded with two different fluorescent protein-tagged molecules or a FRET-based biosensor. As shown in Figure 1A, the *Fluocell* graphic user interface (GUI) recognizes the string patterns of file names recorded in two intensity channels and in a time sequence. The “FRET” or “Ratio” protocol allows the convenient visualization of intensity ratio images (Figure 1A).

The *Fluocell* workflow put an emphasis on processing ratiometric and dynamic image sequences (Figure 1B). The workflow has five steps (Figure 1B): (1) preprocess the images by background subtraction and filtering; (2) visualize the pixelwise ratio images with intensity modulated display (IMD) in 2D and 3D; (3) automatically detect or manually select features/regions of interest (ROIs); (4) track the ROIs and quantify the average ratio signals in time; (5) collect the quantified time courses and perform statistical analysis.

At step (2), a matrix of ratio values was computed in the function *compute_ratio*, where the pixelwise ratio between the images loaded into the first and second channels was calculated with some robust measure. Specifically, for each pixel (*i*, *j*) in the image,

$$ratio\left(i,j\right)=\frac{First\;Channel\;Image\left(i,j\right)+\delta }{Second\;Channel\;Image\left(i,j\right)+\delta },$$

where δ is a small number of the value 1.0e-4. This framework allows the flexibility of choosing any numerator and denominator in ratio calculation, with the goal that the ratio value changes monotonically with and hence represents the targeted molecular activity. Meanwhile, it also allows that FRET efficiency be calculated using other external functions or algorithms and used to replace the current ratiometric calculation in *compute_ratio* (see Supplementary Information for details). The IMD images were calculated in the function *get_imd_image*, by mapping the ratio values to color hue, and the pixelwise average of two intensity images to the brightness, of the HSV color space.

Step (3) provides the option to either simply manually select or automatically detect the ROIs [29]. At step (4), the quantified results can be exported to Excel files for further analysis and interpretation (Figure 1B and the *Fluocell* User’s Guide in Supplementary Materials). Intermediate results in the workflow can be saved and exported from *Fluocell*. For example, the IMD of ratio images can be saved and used to make reports and movies (Figure 1B).

Molecular activities in single cells within a population are heterogeneous and dynamic [30]. To efficiently quantify these dynamic activities for many single cells at subcellular levels, we extended the functionality of *Fluocell* to a multiple-position visualization and analysis module, *Quanty*, which can process multiple dynamic image sequences in a single run (Figure 1C). Briefly, multiple-position imaging data can be collected on a microscope equipped with an automated stage. With the *Quanty* module, the image data can be divided into different subfolders, and then automatically processed to obtain quantified time courses via the *Fluocell* workflow in a single run (Figure 1C). Fluocell is implemented in Java and MATLAB, with its accuracy and computational efficiency carefully characterized (Supplementary Materials, Figures S2, S3).

### Multiple-Sequence/Position Quantification and Visualization of FRET Ratio

**FIGURE 2 |:**
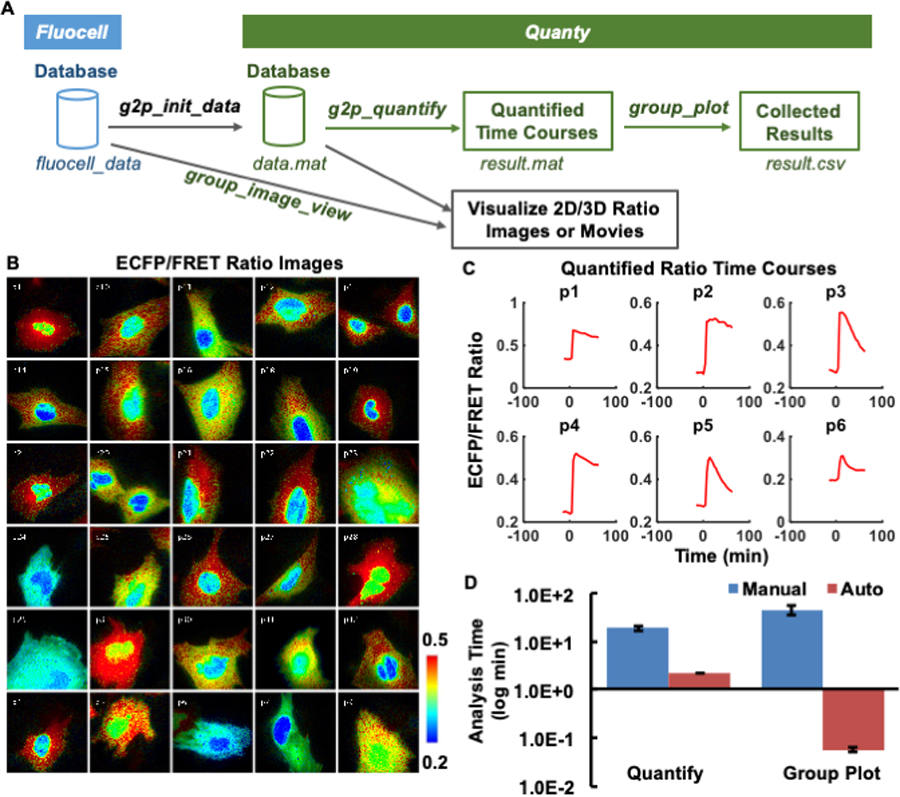
Display of the group view of single-cell ratio images and quantified time courses. **(A)** The schematics showing the interface between *Fluocell* and *Quanty*, as well as the *Quanty* workflow. The quantification and visualization workflow is show in green and back colors, respectively. **(B)** Shows the IMD ECFP/FRET emission intensity ratio images of the cells visualized by the *group_image_view* function. The images are from different positions recorded during the same imaging experiments; **(C)** Shows the emission ratio time courses quantified by the *g2p_quantify* function for the first six cells in the group. **(D)** Compares the required image analysis time between manual analysis by three experienced researchers in *MetaFluor* and automatic analysis by *Fluocell/Quanty* (30 cells, 24 frames/cell). **Left:** time used to quantify the ECFP/FRET ratio time course manually or automatically by *g2p_quantify*; **Right:** time used to plot the time ECFP/FRET ratio time courses based on results from the left panel, manually or automatically by *group_plot*.

Modern microscopes with automated stages allow the collection of live image sequences at multiple spatial positions on the same glass slide (Figure 1C). To visualize and quantify these image sequences at high throughput, we developed the *Quanty* extension package to interface the input data structure with the automatic workflow of *Fluocell* (Figure 2A). Briefly, the *Quanty* functions can be used to calculate multiple time courses from multiple positions, by repeatedly and automatically running *Fluocell* functions on each image sequence (Figure 1C). The functionality of *Quanty* is demonstrated by processing images reported by a new Fyn FRET biosensor, with the ECFP/FRET intensity ratio representing the *in-situ* biosensor phosphorylation mediated by active Fyn kinase [20]. The subcellular biosensor signals are visualized by the IMD display of pixelwise ECFP/FRET ratio, and quantified by the average ECFP/FRET ratio values within the ROIs (Figure 2).

After loading the images into *Fluocell* GUI, all the necessary information was transferred to *Quanty* via the initialization function *g2p_init_data* (Figure 2A). As a result, intensity ratio images of different cells from multiple positions at chosen time points can be visualized by the *group_image_view* function in *Quanty* (Figure 2B and Figure S4). Meanwhile, the average ratio time course of each image sequence can be calculated and plotted by the *g2p_quantify* function (Figure 2C). The average ratio at each time point was evaluated by averaging the pixelwise ratio values within the detected ROIs. At this step, if a quantified time course is under question, it can be backtracked to the corresponding image data, so that the user can screen and control quality of analysis.

**FIGURE 3 |:**
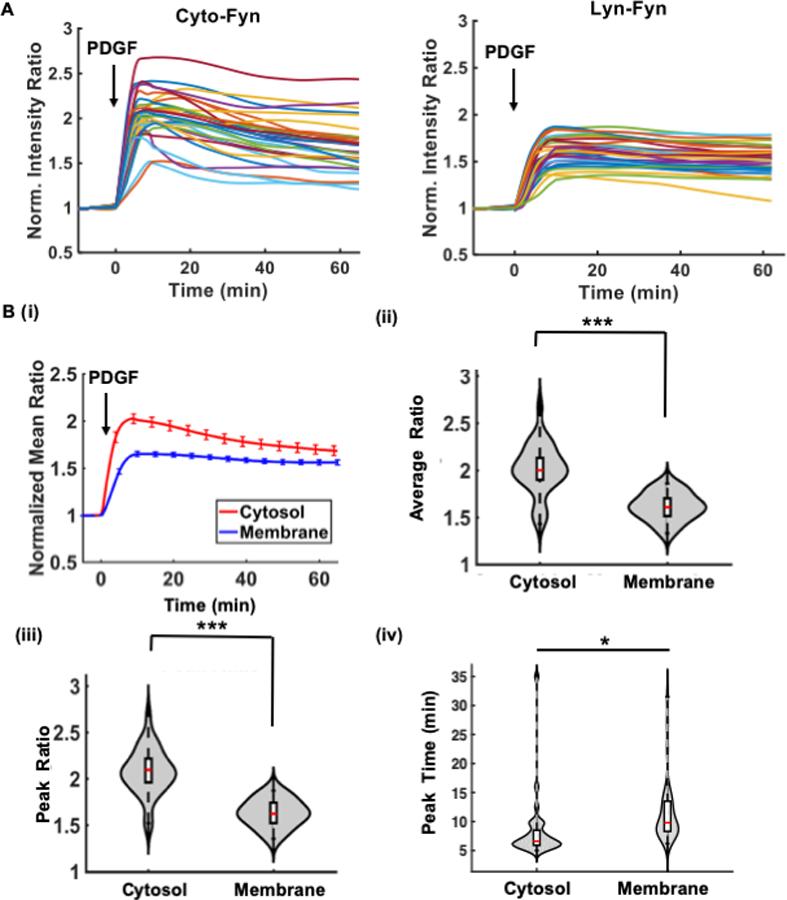
Multiple-cell quantification and statistical interpretation for the cytosolic and membrane-tagged Fyn biosensors in MEF cells. **(A)** The time courses of normalized ECFP/FRET emission ratio of the Fyn biosensor in different cells under growth-factor stimulation. **Left:** the cytosolic Fyn biosensor; **Right:** the membrane-tagged Lyn-Fyn biosensor. **(B)** Statistical comparison of the time courses between the cytosolic and membrane groups: (i) The average time courses of the emission ratio in each group. Error bars: standard error of mean (SEM). Violin plots: (ii) The normalized ratio values averaged between 10 and 20min after PDGF stimulation (10 ng/ µl); (iii) The maximal ratio values; (iv) Time to reach the maximal ratio. *Statistically significant difference, n1 = 29, n2 = 33, *p* < 0.02; ***p < 1.0e-3.

Afterward, the quantified time courses of all cells from multiple image sequences can be plotted together using the *group_plot* function, as well as the average time course with original data points (Figures S5, S6). For normalization, single-cell ratio time courses were divided by its average value before stimulation (basal value) to bring the basal level to 1 across different cells (Figures 3A,B). This technique is often used to allow the comparison of molecular activation kinetics across groups of cells with heterogeneous basal activities. The *Quanty* functions are computationally efficient—increasing the quantification speed by ∼8.6-folds, and that of group statistics by 650-folds when compared with semi-automatic quantification by three experienced scientists using the commercial software package *MetaFluor* (Figure 2D). These results show that *Fluocell* and *Quanty* can automatically process FRET ratio images with objectivity, flexibility, and high efficiency.

The quantified time courses were saved in an Excel file named “result.csv” (on Mac, and “result.xlsx” on MS Windows) in the image data folder. Subsequently the *group_compare* function can be used to compare the statistics of these time courses, such as normalized ratio values averaged among cells in different experimental group, ratio values averaged during a chosen time interval, maximal ratio, and the time to reach the maximal ratio (Figure 3).

### Fyn FRET Ratio Imaging and Statistical Inference

The Src family kinase Fyn plays important roles in cell-matrix interaction, cell migration, and anchorage dependent growth [31, 32]. Our group recently developed a Fyn FRET biosensor to monitor this specific kinase activity in live cells [20]. Mouse embryonic fibroblast (MEF) cells expressing cytosolic or membrane targeted Fyn biosensors were stimulated by platelet-derived growth factor (PDGF) to activate Fyn kinase. Briefly, the cells expressing biosensors were imaged for a few minutes to establish a basal ECFP/FRET ratio value (Figure 3). Microscopic imaging was then paused to allow the addition of PDGF (10µg/ml) into the imaging dish. After resuming imaging, the cells were monitored for about 60min to observe the change of ECFP/FRET ratio images over time, as well as the quantified values within subcellular regions of interest. The FRET ratio images visualized by *Fluocell/Quanty*, with their color changing from blue at 1min after PDGF stimulation to red at 25min, clearly show the activation of Fyn kinase (Figure S4). The image frames between which a stimulation is applied to the cells can be input through the *Fluocell* GUI or via MATLAB, and the time course will be translated such that the time of stimulation is set to 0 (See the *Quanty* User’s Guide for details).

*Quanty* provides some visualization and statistical measures to compare the ratio kinetics between different experimental groups by output in MATLAB command window and visualizing the data distributions via violin plots. Briefly, the *group_compare* function provides statistical visualization with *box_plot* functions, which shows the sample median, 25 and 75 percentiles, and extreme values, and the *violin_plot* function which shows the distribution of data (Figure 3). The function *my_function.statistic_test* function implements MATLAB functions *ttest* for samples of normal distributions, *kstest* for samples of non-normal distributions, and *ranksum* tests for samples of small sizes. In addition, the *multiple_comparison* function provides an interface to the MATLAB *multcompare* with Bonferroni correction.

As shown in Figure 3 and Video S1, quantitative comparison of the biosensor ratio signals indicates that Fyn kinase was activated significantly stronger and faster in the cytosol than the plasma membrane, with a higher average ECFP/FRET ratio in the cytosol during 10–20min after PDGF stimulation, a higher maximal ECFP/FRET ratio value, and shorter time to reach the maximum. The strong cytosolic Fyn kinase signal is probably due to accessibility of the biosensor to active Fyn localized in cytosolic compartments, such as those in centrosomal and mitotic structures near the nucleus [33, 34]. The observed membrane activation can be attributed to the portion of membrane-bound Fyn kinase via myristoylated signals, which can be further affected by the interference of the membrane-targeting motif of the biosensor [33, 35–37]. These results indicate that *Fluocell* and *Quanty* can be applied to efficiently evaluate the dynamic molecular activities in live cells.

### Movie and 3D Visualization

**FIGURE 4 |:**
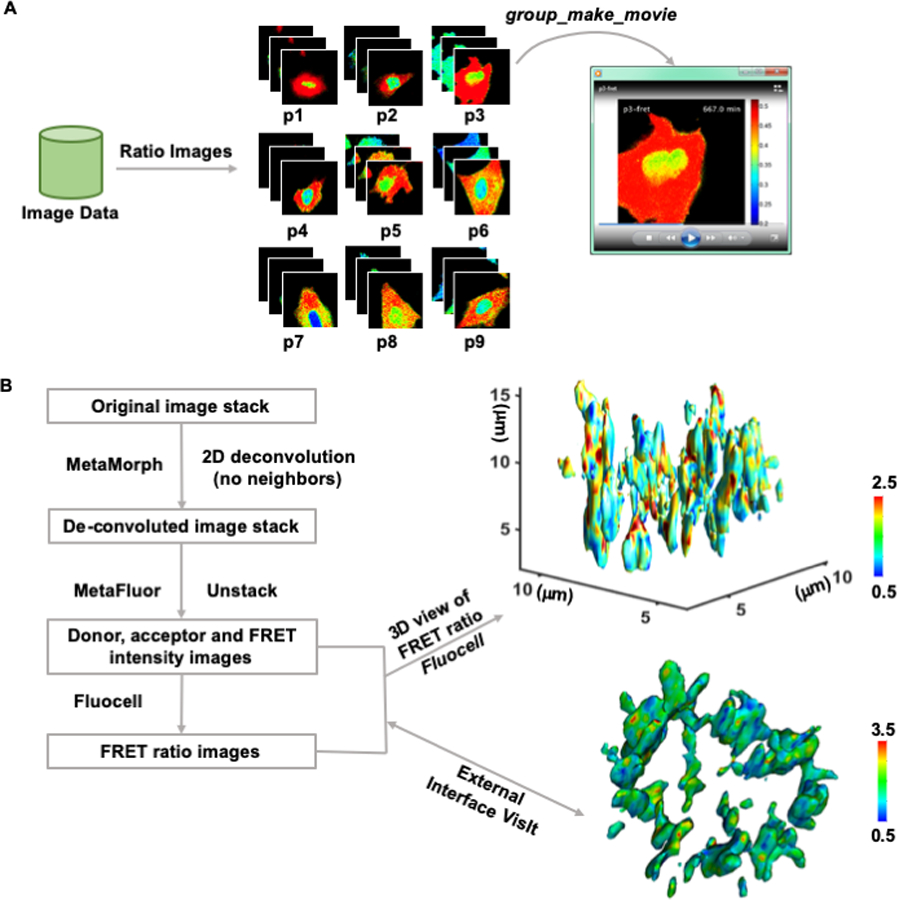
Video and 4D ratio visualization. **(A)** The workflow of *Quanty* in video making. First, raw imaging results are used to generate FRET ratio image sequences at each position. Second, the *group_make_movie* function was used to make a movie at a selected position by connecting the ratio images. The ratio image video of a representative cell with the Fyn biosensor is shown in Video S1. **(B)** The pipeline of 3D ratio visualization. First, MetaMorph and *MetaFluor* were used for 2D deconvolution of the intensity images (detailed in the Supplementary Materials). Then, Fluocell was used to generate Intensity ratio images and provide 3D ratio visualization within MATLAB or through an external software package such as VisIt. The 4D ratio video of a representative cell with the H3K9me3 biosensor is shown in Video S2.

Visualizing live-cell image sequences in movies, z-slices, and the three-dimension space are important for the demonstration and dissemination of experimental results. The *make_movie* and *group_make_movie* functions have been implemented in *Fluocell* and *Quanty,* respectively (Figure 4A and Video S1).

Furthermore, with the input of two sets of fluorescence intensity images, the 2D ratio images can be navigated in z-direction. The *test_3d_view* function provides an interface to the MATLAB function *isosurface*, which can be used to generate a 3D view of the ratio values at a selected intensity isosurface (Figure 4B). The intensity images were pre-processed and de-convoluted in external software packages such as *MetaMorph* and *MetaFluo* (Figure S7). The 3D snapshot is colored by the FRET/ECFP ratio values of a new histone-localized histone 3 lysine 9 tri-methylation (H3K9me3) FRET biosensor at the intensity isosurface of the histone 3 [21]. The color represents level of epigenetic H3K9 tri-methylation at the surface of condensed histone in a dividing HeLa cell. The processed intensity and ratio data can also be exported to allow external 3D visualization in other software packages. For example, to interface with *VisIt* from the Lawrence Livermore National Lab (Figure 4B), the intensity and ratio values in the images were converted to unsigned integers and exported into the red and green component of RGB image files. The 3D rotational views were then generated in *VisIt* and saved to allow further video processing (Figure 4B and Video S2).

The 3D visualization results show discrete hotspots in red color at the surface of condensed histone, which may indicate preserved loci with high H3K9me3 (Figure 4B and Video S2). Since H3K9me3 has been reported to positively regulate closed and protected histone structure, it is possible that the H3K9me3 hotspots can indicate local chromosome regions associated with epigenetic memory containing cell lineage information. Taken together, these results show that *Fluocell* can be used for convenient visualization of ratiometric imaging data to explore dynamic molecular activities in 4D at subcellular levels.

## DISCUSSION

Molecular interactions and functions in live cells are largely dependent on their subcellular location and environment [1, 38]. Molecular activities within a population of cells are heterogeneous and dynamic, with cell-cell variations caused by stochastic subcellular molecular wiring in structure and function [39, 40]. Therefore, accurate and dynamic measurements of molecular activities in live cells often require high-throughput quantification of fluorescence intensity and ratio with subcellular resolution [3, 41]. Furthermore, automated image analysis has the advantage of handing multiplex images from multiple sensors in the same live cells with ease [42].

The quantitative output from image analysis tools can also provide convenient input for the construction of physics-based computational models at subcellular levels [43–45]. It is possible that the single-cell time courses can be used as part of training data for model-informed machine learning algorithms to evaluate reaction kinetic parameters uniformly across all cells, as well as to evaluate molecular concentrations parameters which can adopt different values across cells. Thus, the single-cell time-course data can be used to train a computational model with a distribution of molecular concentration and activity in the modeled network, providing a powerful tool to simultaneously investigate molecular regulation networks and single-cell characteristics. Meanwhile, the kinetic models can also be integrated with biophysical transport models to investigate single-cell molecular regulation with spatiotemporal fidelity to precisely model and to predict cellular and tissue functions [46]. For this purpose, future challenges involve accurately estimating biophysical and biochemical parameter values and distributions based on single-cell spatiotemporal imaging and time-course data, as well as a tight integration between data-driven and model-driven analysis.

The *Fluocell* and *Quanty* software packages were developed for the accurate, efficient, and ratiometric quantification of dynamic image data. For the quantification of FRET dynamics, we utilize simple ratiometric calculation to maximize spatiotemporal resolution of the FRET signal, while minimizing the number of channels imaged to reduce photobleaching, as well as based the three reasons listed below. First, complex algorithms developed previously may provide better accuracy in quantifying FRET efficiency *in vitro* or in a snapshot of cells, but they often require a calibration step to image donor only or acceptor only probes [47, 48]. This calibration can be affected by cellular autofluorescence signals that are intrinsic and variable among different cells or in different subcellular regions. Therefore, it is difficult to utilize these methods for the quantification of subcellular FRET signals in single-live cells. Second, our method directly utilizes images from fluorescence microscopes without the need of switching to lifetime or polarized light microscopy [48, 49]. While the fluorescence lifetime microscopy (FLIM) or polarized microscopy methods may show an advantage in detecting inter-molecular interactions quantitatively, the intensity ratio approach has been widely recognized as a crucial research tool for detecting intra-molecular FRET live-cell studies [50, 51]. Third, the quantified apparent FRET efficiency represents the integral sum of FRET efficiency of biosensors at variable conformation states within the imaged volume, which probably only provides a non-linear measure of the targeting molecular activity and needs further characterization.

Recognizing the variability of FRET signal caused by FP maturation rate [52], expression level, microscope optics, we recommend to use a normalization step to compare the signals before and after signaling events in exactly the same cell and often normalize the signal such that the normalized FRET ratio time courses show a relative change from a basal level of 1 (Figure 3) [6, 23, 40, 53, 54]. The normalization step allows comparing samples across different experiments performed on the same or potentially different microscopes with distinct optical settings. The normalized ratiometric readout, in turn, can allow the experimental data acquisition and analysis to be performed in parallel in many bioimaging and biotechnology laboratories. During imaging, we also attenuate the strength of excitation (with neutral density filters) and limit the exposure time and frequency of fluorescence sample, such that significant effect of photobleaching was not observed in the control time course before signaling with our imaging protocol (Figure S3B and Supplementary Materials) [20, 55].

Utilizing this imaging and analysis protocols, our group has published an array of papers engineering FRET biosensors and quantifying the time courses of dynamic molecular activities for kinases, proteases, and membrane channels in live cells [6, 23, 24, 26, 28, 40, 53, 54, 56]. Currently, we do not correct for multi-channel cross-talks and bleed-through between fluorophores or estimate pixelwise FRET efficiency by default [57]. On the other hand, alternative quantification methods can provide a preferred measurement of FRET signal or molecular activity under certain conditions. Therefore, a user interface is provided to allow any user supplied function to be used for calculating FRET signal (see Supplementary Information on *compute_ratio*), with the designation that our software package can contribute to the imaging and analysis communities and help further the goal of quantifying molecular activity dynamics in single live cells.

Both *Fluocell* and *Quanty* have modularized design, to be used alone or in combination with other image analysis tools. *Fluocell* and *Quanty* are suitable for images with high spatiotemporal resolution, which allow the detection of subcellular dynamic events such as epigenetic modification at important DNA loci and the assembly and dissolution of focal adhesions with accuracy [58, 59]. Since the size of image data is usually big, *Fluocell* and *Quanty* normally run on a computer with local access to data. When the data size is relatively small, the data can also be transferred via internet or accessed remotely by the software packages. Our results indicate the software packages can significantly improve the efficiency of biological workflow, and hence provide valuable tools for single-cell analysis [8, 60, 61].

Ratiometric visualization and quantification of imaging data for FRET biosensors indicate that Fyn kinase was activated faster and stronger in the cytoplasm of MEF cells stimulated by PDGF. It is possible that more activatable Fyn kinases are located at the perinuclear regions of the cytoplasm. In addition, Fyn kinase can promote phosphorylation of the transmembrane adaptor molecule PAG, which recruits Csk, a known inhibitor of Fyn [62]. Therefore, at cell membrane, Fyn kinase activation can trigger a negative feedback loop to modulate its own activity, which may contribute to the relatively low membrane Fyn signal observed by our biosensor. On the other hand, our H3K9 tri-methylation results show 3D hotspots of high H3K9me3 levels at the surface of condensed chromosomes, with important lineage preserving implications. Thus, *Fluocell* provides an efficient and convenient tool to quantitatively compare and visualize dynamic ratiometric imaging results at the single-cell level to provide biologically significant results.

## Supplementary Material

Supplementary Info

Video S1

Video S2

## Abbreviations:

FRET: Förster or fluorescence, resonance energy transfer
GUI: graphic user interface
ECFP: enhanced cyan fluorescent protein
YPet: yellow fluorescent protein
IMD: intensity modulated display
ROIs: regions of interest
SEM: standard error of mean
PDGF: platelet-derived growth factor
