# Supplementary material for: Fluocell for Ratiometric and High-Throughput Live-Cell Image Visualization and Quantitation
Source: Front Phys. Author manuscript; Available in PMC 2020 Nov 6. (PMC7646842; doi:10.3389/fphy.2019.00154)
Supplement: Supplementary Info [file NIHMS1568048-supplement-Supplementary_Info.pdf]

Fluocell for Ratiometric and High-throughput Live-cell Image Visualization and Quantitation

Qin Qin[1], Shannon Laub[1], Yiwen Shi[2], Mingxing Ouyang[1], Qin Peng[1], Jin Zhang[3], Yingxiao Wang[1], Shaoying Lu[1,2,*]

[1]Department of Bioengineering, Institute of Engineering in Medicine, [2]Department of Mathematics, Center of Computational Mathematics, [3]Department of Pharmacology, University of California, San Diego, La Jolla, California, USA.

* Correspondence should be addressed to:

Shaoying Lu: kalu@ucsd.edu

Appendix and Supplementary Materials

Software implementation

The *Fluocell* GUI is implemented in Java and connected with image analysis and visualization functions in MATLAB, with the image data being input from any location on a local computer (Fig. S2). The source code can also be run by a programmer in the MATLAB command window. Java was chosen for the convenient implementation of GUI with the NetBean IDE; MATLAB was preferred for analysis because of its optimized image/matrix operations and advanced Image Processing and Statistical Analysis toolboxes. Both programming languages are cross-platform compatible, and so are *Fluocell* and *Quanty*. The main programming structure and batch processing workflow are shown in Figures S2B-S2C. The complete workflow can be tested by running the test functions *test_fluocell* and *test_quanty* with the included dataset. Additional functions and data sets are provided in the folder *"quanty/app/test/"* to repeat the results shown in the figures.

The accuracy and efficiency of the spatiotemporal quantification functionality of *Fluocell*

To automatically process image data from multiple video sequences, it is important to automate every step in the quantification workflow. During pre-processing, we implemented an option to automatically detect and subtract background regions. Briefly, among four corners of the image, the square with the smallest average fluorescence intensity was automatically selected as the background region (Figs. S3A). The average intensity values of the background region were then calculated as the ambient background and subtracted from the corresponding intensity images before calculating pixelwise ratio (Figs. S3A and S3B). As shown in Figure S3B, automatic background selection produces a quantified time course of similar accuracy as manual selection. Furthermore, the ECFP and FRET intensity values remain relatively constant for ~10 min before stimulation, and no obvious photobleach was been observed in either channel. As a result, the ratio values also remained within

5% of its initial value at 0 min. This result confirms that no significant photobleach was observed using our imaging protocol.

For subcellular quantification, Fluocell can automatically track manually selected ROIs over time according to their relative distance and orientation to the cell centroid. The quantified time course has an accuracy similar to that obtained with relatively tedious manual correction (Figs. S3C and S3D). Alternatively, the cell body can be automatically detected and divide into different subcellular regions according to their relative distance to the extracellular space, by the Fluocell function *divide_layer* which interfaces the MATLAB function *bwdist* function and Euclidian distance transformation (Fig. S3E) (Maurer, et al., 2003). Thus, automatically selected subcellular ROIs naturally follow the nonlinear movement of a cell and allow the quantification of a smooth time course (Figs. S3E and S3F). In addition, the imaging time was automatically extracted from the original files via the *get_time* function (Fig. S3G). Taken together, these results show that *Fluocell* allows accurate and automatic quantification of dynamic ratiometric signals with subcellular resolutions.

For both membrane and cytoplasmic sensors quantified in Figure 3, the imaging focus plan was chosen at the bottom plasma membrane of the cell near the cover glass on the inverted widefield microscope. The signals of the cytoplasmic biosensor represent a projection of the fluorescence signal from the 3D focal space onto 2D images. With ratiometric calculation, the quantified time courses are relatively independent of the levels of sensor expression and effect of projection. As for the membrane localized Lyn-Fyn biosensor, majority of the quantified signals should come from the bottom plasma membrane, the signals from internal membrane of intracellular organelles can contribute only if they are also near the bottom of cells. In this case,

other image modality such as confocal, or 3D deconvolution-based approaches be used to further analyze FRET at subcellular locations.

To attenuate the effect of the nucleus or perinuclear organelles on the ratio signals, the cell mask was divided into 3 layers using the Fluocell function *divide_layer*, while the average ratio values were computed from the pixels in the outermost layer (Fig. 2C and the "Auto-1" region in Fig. S3E). Thus, the quantified ECFP/FRET ratio time courses represent the activity of Fyn kinase near the cell peripheral. This region of interest was chosen to quantify all the time courses shown in Figures 2 and 3. The option can be turned on in *quanty* by setting:

```
>> data.quantify_roi = 3; % tracking cell body
% divide cell into 3 layers and quantify the outer layer (Auto-1)
>> data.num_roi = 3;
```

To quantify the dynamic intensity ratio for multiple cells in the same image sequence, the class *multiple_object* was developed with multiple-cell tracking functions. The *multiple_object* class interfaces *Fluocell* with the MATLAB file exchange function, *simpletracker*, to dynamically link cells across time based on the spatial location of their centroids (Figs. S3G and S3H). With the *multiple_object* class, *Floucell* can track multiple cells, detect splitting events, and quantify ratio signals over time in the imaging sequences (Figs. S3H and S3I). The Hungarian particle linking algorithm based on the Munkres Assignment was used for tracking cells (Burgeois and J.-C., 1971). This version of nearest neighbor linking algorithm was implemented in a MATALAB simpletracker function by Jean-Yves Tinevez, and characterization of the performance is available at MathWorks File Exchange site. This method is good for tracking cells in a relatively sparse setting where the cells were kept at a good distance from each other and their paths do not cross between two consecutive

time frames. The method is applicable when the cells are in relatively sparse culture or when expression efficiency is relatively low, not designed to handle dense cell culture in the current form. A splitting event is flagged when a new track is initiated near mother cell in an existing tracking, and when the size of the mother cell shrank 20% or more during 2-3 frames before splitting (indicating the mother cell is rounding up and ready to split). Correct tracking requires that the roundup event be imaged at least twice before a cell splits, or the same cells need to be imaged in a time interval of 10 min or less. The fluorescence images were used to calculate the size of cells.

Supplementary Materials and Methods

Image Analysis

All the intensity images can be preprocessed with two optional steps: background subtraction to cancel background illumination and median filtering with 3×3 pixel window size to remove single-pixel noise. With the Fyn biosensor, after combining the biosensor's ECFP and FRET images, *Fluocell* calculates the cell mask with global image threshold using Otsu's method (Otsu, 1979). To attenuate the effect of the nucleus or perinuclear organelles on the ratio signal, the cell mask was divided into 3 layers using the Fluocell function *divide_layer*, while the average ratio values were computed from the pixels in the outermost layer (Figs. 2C, S3E, and S3F). The *divide_layer* function interfaces with MATLAB function *bwdist* to divide a cell mask into *n* layers according to the distant from a pixel to the background region outside of the cell (Wu, et al., 2016). Therefore, the quantified ECFP/FRET ratio time courses represent the activity of Fyn kinase near the cell peripheral.

During mammalian cell mitosis, the chromosomes compact, and the nucleus-localized H3K9me3 biosensors condense into discrete 3D structures. Under a widefield fluorescence microscope, the biosensor emission will show up as a stack of hazed images (Fig. S7). Therefore, a deconvolution step is needed to reduce the effect of out-of-focus haze during image processing

(Schneider, et al., 2012). Specifically, metamorph 2D "no neighbor" deconvolution (not considering out-of-focus information contained in adjacent planes), with a filter size of 9 and option to suppress noise, was used to reduce the effect of out-of-focus haze from a stack of images. Identical deconvolution algorithms were applied on the ECFP and FRET intensity images before subtracting background and calculating pixelwise ratio. As a result, 4D sub-chromosomal H3K9me3 structure and methylation modifications became significantly sharpened (Figs. S7 and 4B). It is possible that deconvolution may influence both the intensity and ratiometric measurements, so future characterization and improvement on this optional pre-processing step is needed. Deconvolution should only be used for nucleus-localized biosensors such as our H3K9me3 biosensor in mitotic cells, but not in interface cells or for cytoplasmic biosensors or fluorescence proteins tags.

For additional details on the usage of Fluocell/Quanty, please refer to the Fluocell and Quanty User's Guide.

Background Subtraction Options

The automatic option for background subtraction is based on the average intensity of the darkest patch in one of the four corners in the images. To deal with the situation when each corner has a cell in it, we implemented additional options for background subtraction. For example, the background value can be chosen as a given percentile of the intensity image histogram. The value of the percentile can also be altered depending on the histogram of image intensity. The option can be turned on by setting

```
>> fluocell_data.subtrack_background = 4;
```

Alternatively, a flatfield correction can be enabled when a background image is read from the TIFF image file output/background.tif for correction. This option can be turned on by setting

```
>> fluocell_data.subtrack_background = 5;
```

When a reference flatfield correction is not available, a high pass filter can be turned on to allow the subtraction of low-variation background. The value of high_pass_filter is chosen to be an odd integer number much bigger than the estimated diameter of object detected and smaller than the image size. For example, it can be turned on by setting

```
>> fluocell_data.high_pass_filter = 61;
```

Finally, there is also an option in the Fluocell Menu for “Subtract Background” which gives the user the option to select “1 – Manual.” This option allows the user to choose a small region of the image for background subtraction.

Code Availability

The *Fluocell* and *Quanty* software packages are distributed under the GNU Lesser General Public License (version 2.9), the source code and C++ library of this customized FRET image analysis system can be downloaded and modified freely for research usage from our group website (http://wang.ucsd.edu/~kalu/fluocell_paper_2017) and GitHub (http://github.com/lu6007/fluocell) for the MS Windows and Mac operating systems.

Code Segments

The code segment in the *test_3d_view* function is shown below to demonstrate the image files are loaded and processed in the Fluocell environment for the visualization of ratiometric image z-stacks, as well as how the data was parsed to interface with *VisIt*.

```
%% load images in time frames
temp = imread(data.first_file);
im = preprocess(temp, data); clear temp;
figure; imagesc(im);
rect = data.rectangle;
nsize = floor([rect(4); rect(3)]+0.5);
num_frame = length(image_index);
fret_im = zeros(nsize(1), nsize(2), num_frame);
cfp_im = zeros(size(fret_im));

for i = 1:num_frame
    j = image_index(i);
    j_str = sprintf(data.index_pattern{2}, j);
    file_name = regexprep(data.first_file, data.index_pattern{1}, j_str);
    temp = imread(file_name);
    fret_im(:,:,i) = preprocess(temp, data);
    clear temp; temp = file_name; clear file_name;
    file_name = regexprep(temp, data.channel_pattern{1}, data.channel_pattern{2});
    clear temp; temp = imread(file_name);
    cfp_im(:,:,i) = preprocess(temp, data);
    clear j_str temp file_name;
end
figure; imagesc(fret_im(:,:,8));

% Compute ratio and intensity images
ratio_im = compute_ratio(fret_im, cfp_im, 'shift', intensity_base);
intensity_im = 1/(1+ratio_factor)*fret_im+ratio_factor/(1+ratio_factor)*cfp_im;
intensity_bound = [1 1023];

%% output files for 3d view in visIt
for i = 1:num_frame
    j = image_index(i);
    j_str = sprintf(data.index_pattern{2},j);
    file_name = strcat(p, 'output/im_rgb_',j_str,'.tiff');
    temp = floor(0.5+imscale(intensity_im(:,:,i), 1, 255, intensity_bound));
    im_red = ind2rgb(temp, gray(255)); clear temp;
    temp = floor(0.5+imscale(ratio_im(:,:,1), 1, 255, ratio_bound));
    im_green = ind2rgb(temp, gray(255)); clear temp;
    im_blue = double(i/255)*ones(size(im_green));
    im_rgb = zeros(size(im_red));
    im_rgb(:,:,1) = im_red(:,:,1);
    im_rgb(:,:,2) = im_green(:,:,2);
    im_rgb(:,:,3) = im_blue(:,:,3);
    imwrite(im_rgb, file_name, 'tiff', 'Compression', 'none');
    clear j_str file_name im_red im_green im_blue im_rgb;
end
disp('red - intensity; green - ratio; blue - z_index');
disp(strcat('z_dist = ', num2str(z_dist), '; 15 pixels - 1 um'));
disp(strcat('intensity_bound = ', num2str(intensity_bound)));
disp(strcat('ratio_bound = ', num2str(ratio_bound)));

%% Calculate visualize the ISO surface
% 15 pixel/micron in z-plane
[xx, yy, zz] = meshgrid(1:nsize(2), 1:nsize(1), (1:num_frame)*z_dist);
screen_size = get(0, 'ScreenSize');
fig = figure('Position', [50 50 screen_size(4) screen_size(4)], ...
    'color', 'w');
```

```
isosurface(xx, yy, zz, intensity_im, iso_value, ratio_im);
caxis(ratio_bound); shading interp; colormap jet;
axis tight; xlabel('x-pixel'); ylabel('y-pixel'); zlabel('z-pixel');
camlight right;

fig = figure('Position', [50 50 screen_size(4) screen_size(4)], ...
    'color', 'w');
isosurface(xx/15, yy/15, zz/15, intensity_im, iso_value, ratio_im);
caxis(ratio_bound); shading interp; colormap jet;
axis tight; xlabel('x-\mu m'); ylabel('y-\mu m'); zlabel('z-\mu m');
set(gca, 'FontSize', 24, 'FontWeight', 'Bold');
set(gca, 'LineWidth', 3);
camlight right;
```

The compute_ratio function is used to calculate the numerator/denominator ratio while protect against zero division. It can also be customized and replaced by any user supplied function for calculating FRET efficiency by setting

>> fluocell_data.compute_ratio_function = @user_supplied_function

```
% function ratio = compute_ratio(top, bottom);
% Compute the ratio image between images top and bottom with a
% protection against zero denominator.
%
% parameter_name = {'shift', 'method'};
% default_value = {1.0e-4, 1};
%
% method --- 1: increment both top and bottom by the value of 'shift'
% method --- 2: only when the bottom entry is less than shift, increment by
shift
% When the bottom entry is less than 1.0e-4, increment it by 1.0e-4

% Copyright: Shaoying Lu and Yingxiao Wang 2011
function ratio = compute_ratio(top, bottom,varargin)
parameter_name = {'shift', 'method'};
default_value = {1.0e-4, 1};
[shift, method] = parse_parameter(parameter_name, default_value, varargin);
if method ==1
    % increment both top and bottom by the value of 'shift'
    ratio = (double(top)+shift)./(double(bottom)+shift);
else % method ==2,
    % only when the bottom entry is less than shift, increment by shift.
    ratio = double(top)./(double(bottom)+double(bottom<shift)*shift);
end
return;
```

DNA constructs

The sequences and construction methods for the enhanced cyan fluorescent protein (ECFP) and yellow fluorescent protein (YPet) was previously described (Lu, et al., 2008).

The Fyn biosensor was first constructed into the vector pRSETb for bacterial expression. The DNA fragment including SH2 domain, a 15 amino acids linker and the substrate peptide EKIEGTYGVV was amplified by PCR based on the template of a Src biosensor (Lu, et al., 2008). The PCR fragment with SH2 domain containing a single-site mutation (C185A) or triple-site mutation was amplified similarly from two newly improved Src biosensors as the template (manuscript in preparation). The individual PCR fragment was ligated between ECFP and YPet fluorescent proteins from the Src biosensor by SphI/SacI restriction sites. The biosensors with the three versions of SH2 are the same except for the mutations in SH2 domain. The biosensor in pRSETb was further amplified by PCR and ligated into the mammalian expression vector pCAGGS (Komatsu, et al., 2011) by using EcoRI/SalI sites. The negative Y/F mutation in the substrate of the biosensor was introduced by site-directed mutagenesis (following the protocol from Agilent Technologies) in the pRSETb version, and the mutated biosensor was further subcloned into pCAGGS vector. The membrane-targeted biosensors were constructed by PCR amplification of the biosensor while adding the Lyn tag (MGCIKSKRKDNLNDDE), the longer Lyn tag with 21aa (MGCIKSKRKDNLNDDGVDMKT) at the N-terminus of the biosensor, and then by ligation into pCAGGS vector with EcoRI/SalI sites.

For the H3K9me3 biosensor, the cDNA of C-terminal truncated YPet (TYpet) and LacZ were generated using PCR and that of full-length histone H3 was synthesized by gBlocks from IDT. All of three fragments were cloned into pCBI vector, which has a mutated BsaI site to allow convenient cloning strategies, based on the original pCAGGS vector (Komatsu, et al., 2011). HP1 was a gift from Alice Ting (Addgene plasmid #22866). The cDNA of HP1 and ECFP were generated using PCR, and EV linker was synthesized by gblocks from IDT. Three fragments were cloned into

pCBI-TYPet-LacZ-H3 construct to generate wild-type H3K9me3 FRET biosensor by Golden Gate Assembly (Engler, et al., 2009; Engler, et al., 2008; Engler and Marillonnet, 2011). The wild-type H3K9me3 biosensor was transferred into lentiviral transfer vector pSIN by using Gibson Assembly Master Kit (New England BioLabs). W45A, K9L, S10A, and S10E mutants were created by generating mutations on HP1 and histone H3, respectively, in WT H3K9me3 biosensor plasmid using the QuickChange Site-Directed Mutagenesis Kit (Agilent Technologies). Replacement of HP1 domain with SUV39H1 was carried out using Golden Gate Assembly to generate a methyltransferase SUV39H1 FRET biosensor. Replacement of ECFP/YPet FRET pair in WT H3K9me3 biosensor with LSSmOrange/FusionRed FRET pair (Shcherbakova, et al., 2012; Shemiakina, et al., 2012) was carried out using Golden Gate Assembly to generate red fluorescent protein based H3K9me3 biosensor. The FHA2 version phosphorylation biosensor was generated by modifying the aurora B sensor (Fuller, et al., 2008). The aurora B sensor was a gift from Michael Lampson (Addgene plasmid #45214). The substrate sequence was replaced with ARAKQAARK<u>S</u>AGGK (H3-3TM) or ARAKQAARK<u>A</u>AGGK (H3-S3TM). For targeting the H3S10p biosensor to nucleosome, a full-length histone H3 was inserted in place of CENP-B. pCBI-mCherry-H3.3 WT and pCBI-mCherry-H3.3K9M were constructed by Golden Gate Assembly, based on the parental template of H3F3A gene. H3F3A was a gift from Bing Zhu (Addgene plasmid #47981). pLL3.7m-mOrange2-SLBP(18-126)-IRES-H1-mMaroon1 was constructed by Gibson Assembly, and the template of SLBP(18-126)-IRES-H1-mMaroon1 was a gift from Michael Lin (Addgene plasmid #83842).

Cell Culture, Transient Transfection, and Reagents

All cell lines were obtained from ATCC and cultured in the incubators at 37°C, supplemented with humidified 95% air and 5% CO_2. MEFs and HeLa cells were cultured in Dulbecco's modified Eagle's medium (DMEM) supplemented with 10% fetal bovine serum, 2 mM L-glutamine, 100

unit/ml penicillin, 100 µg/ml streptomycin, and 1 mM sodium pyruvate (GIBCO BRL) before transfection. The cell culture reagents were obtained from Life Technologies (Gibco).

Fyn biosensor DNA plasmids were transfected into MEF cells by using Lipofectamine 2000 according to the protocol from the vendor (Invitrogen). The transfection condition was optimized for MEFs, with 3 µg of DNA plasmids for each small cell culture dish (35 mm in diameter). If the cell density was high (e.g. >80% confluency), 4 µg of DNA plasmids were applied for each dish. HeLa cells were transfected in separate experiments with DNA plasmids containing sequences for the H3K9 biosensors. Cells were passed on fibronectin-coated micropatterned glass surface for 2–6 hrs prior to imaging. To avoid the detrimental effects of trypsin on cell migration when passing cells, cells were detached by a mild 4 mM EDTA (pH 7.4, incubation at 37°C for 8–10 min) in PBS.

Microscope Image Acquisition

During imaging, cells were cultured in cover glass bottom dishes (Cell E&G) and maintained in CO_2-independent medium (Life Technologies/Gibco) without serum at 37°C. Images were collected by a Nikon Eclipse Ti inverted microscope equipped with 40x objective (NA 1.30) or 100x objective (NA:1.45) and a cooled charge-coupled device camera (Cascade 512B; Photometrics) using the *Metamorph/MetaFluor* 7.8 software (Molecular Devices). The images were collected with a 420DF20 excitation filter, a 450DRLP dichroic mirror, and two emission filters controlled by a filter changer (475DF40 for ECFP and 535DF25 for FRET). To avoid photobleach during imaging, neutral density blocks were used to reduce the excitation strength to 1/32-1/8 of the full power. In addition, the exposure time was set to about 800 ns for each ECFP or FRET emission image acquired. The frequency of image time points was also limited to no

more than 2 time points each minute. The pixelwise images of ECFP/FRET or FRET/ECFP emission ratio were then computed to assess the FRET signals using *Fluocell* and *Quanty*.

~~Author Contributions~~

~~Q.Q. and S.Laub authored the software, performed image analysis, and wrote the manuscript, Y.S. authored the software, M.O. and Q.P. contributed the FRET biosensor image data and performed analysis, Y.W. conceived the idea and wrote the manuscript, S.Lu. conceived the idea, authored the software and manuscript, and performed image analysis.~~

Acknowledgements

This work was supported by the National Science Foundation under NSF/NIH Math/Bio Initiative DMS-1361421 (S.Lu, Q.Q., and Y.Wu), the National Institute of Health HL121365 (S.Lu and Y.Wang), and UC FISP undergraduate research fellowship (S.Laub).

Supplementary Figure Legends

Supplementary Figure S1. The FRET donor and acceptor spectra and working principle. **(A)** shows the excitation and the emission spectra of the donor, enhanced cyan fluorescence protein (ECFP) and the acceptor, a yellow fluorescence protein (YPet) obtained from the spectra database hosted at the University of Arizona. **(B)** shows different conformations of a single-molecular FRET biosensor with (left) or without (right) energy transfer.

Supplementary Figure S2. The main programming structure and workflow of *Fluocell*. (A) The schematics showing the interaction between image data, graphic user interface (GUI), and visualization and quantification function. **(B)** shows the organization of source code and files in subfolders of Fluocell. Majority of source codes are in the "src" folder, while the "app" and "doc" folders hold test functions and documentation respectively. The subfolders in "src" are "pre" for pre-processing, "vis" for visualization, "api" for programming interface, "gui" for graphic user interface, "detect" for detection, "track" for tracking, and "post" for post-processing. **(C)** shows the workflow for visualizing and quantifying ratiometric images. The *update_ratio_image* function is the main internal function for ratiometric image visualization and quantification, while the *get_imd_image* function generate the IMD image based on an input intensity image and a ratio image.

Supplementary Figure S3. Characterize the accuracy of *Fluocell* analysis. (A) The IMD of ECFP/FRET ratio images in a cell are shown with background regions outlined in yellow (lower left corner). The background regions were either manually selected by the user (top), or automatically generated (bottom) by *Fluocell*. **(B)** The left panel compares the time courses of ECFP/FRET ratio averaged in the whole cell, based on the background region chosen in (A). Right panel: in the same cell shown in the left pane, the quantified values of ECFP and FRET intensity, and ECFP/FRET ratio normalized to those at 0 min are plotted vs. time. **(C)** The ECFP/FRET ratio images of a moving cell are shown with different ROIs outlined in solid white. **Left:** The initial ROI specified by user; **middle:** the same fixed ROI at 32 min when the cell moved; **right:** the tracked ROI at 32 min in this cell; **(D)** Compare the time courses of ECFP/FRET ratio averages in the ROIs shown in (C). **(E)** The ECFP/FRET ratio images of a cell is overlaid with regions of interest (ROIs). The ROIs outlined in solid red lines and indexed in yellow were either manually selected by user (left) or automatically selected according to its distance to the extracellular space (right); **(F)** Compare the time courses of ECFP/FRET ratio averages in the ROIs shown in (E). The corrected time course was generated by manually moving the ROI which roughly follows the movement of the cell. **(G)** Automatically retrieve the time of imaging by the *get_time* function. In this function, the output of the MATLAB function *imfinfo* was used to calculate the actual imaging time in minutes from 12 am of the day when the images were first saved. **(H)** Ratio images overlaid with detected cell edge and indexed with track number. **(I)** Time course of ratio values quantified from the image sequence shown in (H). Track 1 split into tracks 1 and 3 since the cell divided.

Supplementary Figure S4. The ECFP/FRET ratio images of the cells at other time points. The intensity ratio images of the cells acquired at multiple positions at **(left)** 1 min or **(right)** 25

min after PDGF stimulation. The ratio color changes from cold to hot, representing low and high ECFP/FRET values.

Supplementary Figure S5. The ECFP/FRET ratio image and original time courses of the cytosolic Fyn FRET biosensor. The ECFP/FRET ratio images of the cytosolic Fyn FRET biosensor in the same single MEF cells are compared **(A)** before and **(B)** after PDGF stimulation. **(C)** The quantified time courses of the cytosolic ECFP/FRET ratio in cells shown in (A) and (B). Each curve represents a time course from a single cell. **(D)** The distribution of intensity ratio time courses in (C) (light gray circles) are plotted with the average time course. Error bars: Mean±SEM.

Supplementary Figure S6. The ECFP/FRET ratio image and original time courses of the membrane tethered Lyn-Fyn FRET biosensor. The ECFP/FRET ratio images of the Lyn-Fyn biosensor in the same single MEF cells are compared **(A)** before and **(B)** after PDGF stimulation. **(C)** The quantified time courses of the Lyn-Fyn ECFP/FRET ratio in cells shown in (A) and (B). Each curve represents a time course from a single cell. **(D)** The distribution of intensity ratio time courses in (C) (light gray circles) are plotted with the average time course. Error bars: Mean±SEM.

Supplementary Figure S7. The effect of de-convolution in processing 3D image slices during cell mitosis. Top panels show the ECFP (left), FRET (middle) and the IMD FRET/ECFP ratio (right) images of a representative z-slice in a 3D image stack. Lower panels show the corresponding images pre-processed by deconvolution.

Supplementary Video Legends

Supplementary Video S1. Fyn activation by PDGF in a live MEF cell. The ECFP/FRET ratio images of a live MEF cell changed color from cold (blue) to hot (red) when it was treated by PDGF, indicating the activation of the Src family kinase Fyn visualized by a Fyn FRET biosensor.

Supplementary Video S2. 4D H3K9 tri-methylation distribution at the surface of condensed chromosome during mitosis. The rotational 4D view of H3K9me3 FRET/ECFP ratio video at the surface of the chromosomal structure of a live HeLa cell in mitosis. The video shows the rotational view of the chromosome surface at six time points during 0-25 min of imaging.

Supplementary References

Burgeois, F. and J.-C., L. An extension of the Munkres algorithm for the assignment problem to rectangular matrices. *Communications of the ACM* 1971.

Engler, C.*, et al.* Golden gate shuffling: a one-pot DNA shuffling method based on type IIs restriction enzymes. *PLoS One* 2009;4(5):e5553.

Engler, C., Kandzia, R. and Marillonnet, S. A one pot, one step, precision cloning method with high throughput capability. *PLoS One* 2008;3(11):e3647.

Engler, C. and Marillonnet, S. Generation of families of construct variants using golden gate shuffling. *Methods Mol Biol* 2011;729:167-181.

Fuller, B.G.*, et al.* Midzone activation of aurora B in anaphase produces an intracellular phosphorylation gradient. *Nature* 2008;453(7198):1132-1136.

Komatsu, N.*, et al.* Development of an optimized backbone of FRET biosensors for kinases and GTPases. *Mol Biol Cell* 2011;22(23):4647-4656.

Lu, S.*, et al.* The spatiotemporal pattern of Src activation at lipid rafts revealed by diffusion-corrected FRET imaging. *PLoS Comput Biol* 2008;4(7):e1000127.

Maurer, C.R., Qi, R.S. and Raghavan, V. A linear time algorithm for computing exact Euclidean distance transforms of binary images in arbitrary dimensions. *Ieee T Pattern Anal* 2003;25(2):265-270.

Otsu, N. Threshold Selection Method from Gray-Level Histograms. *Ieee T Syst Man Cyb* 1979;9(1):62-66.

Schneider, C.A., Rasband, W.S. and Eliceiri, K.W. NIH Image to ImageJ: 25 years of image analysis. *Nat Methods* 2012;9(7):671-675.

Shcherbakova, D.M.*, et al.* An orange fluorescent protein with a large Stokes shift for single-excitation multicolor FCCS and FRET imaging. *Journal of the American Chemical Society* 2012;134(18):7913-7923.

Shemiakina, II*, et al.* A monomeric red fluorescent protein with low cytotoxicity. *Nat Commun* 2012;3:1204.

Wu, Y.*, et al.* In-situ coupling between kinase activities and protein dynamics within single focal adhesions. *Scientific reports* 2016;6:29377.

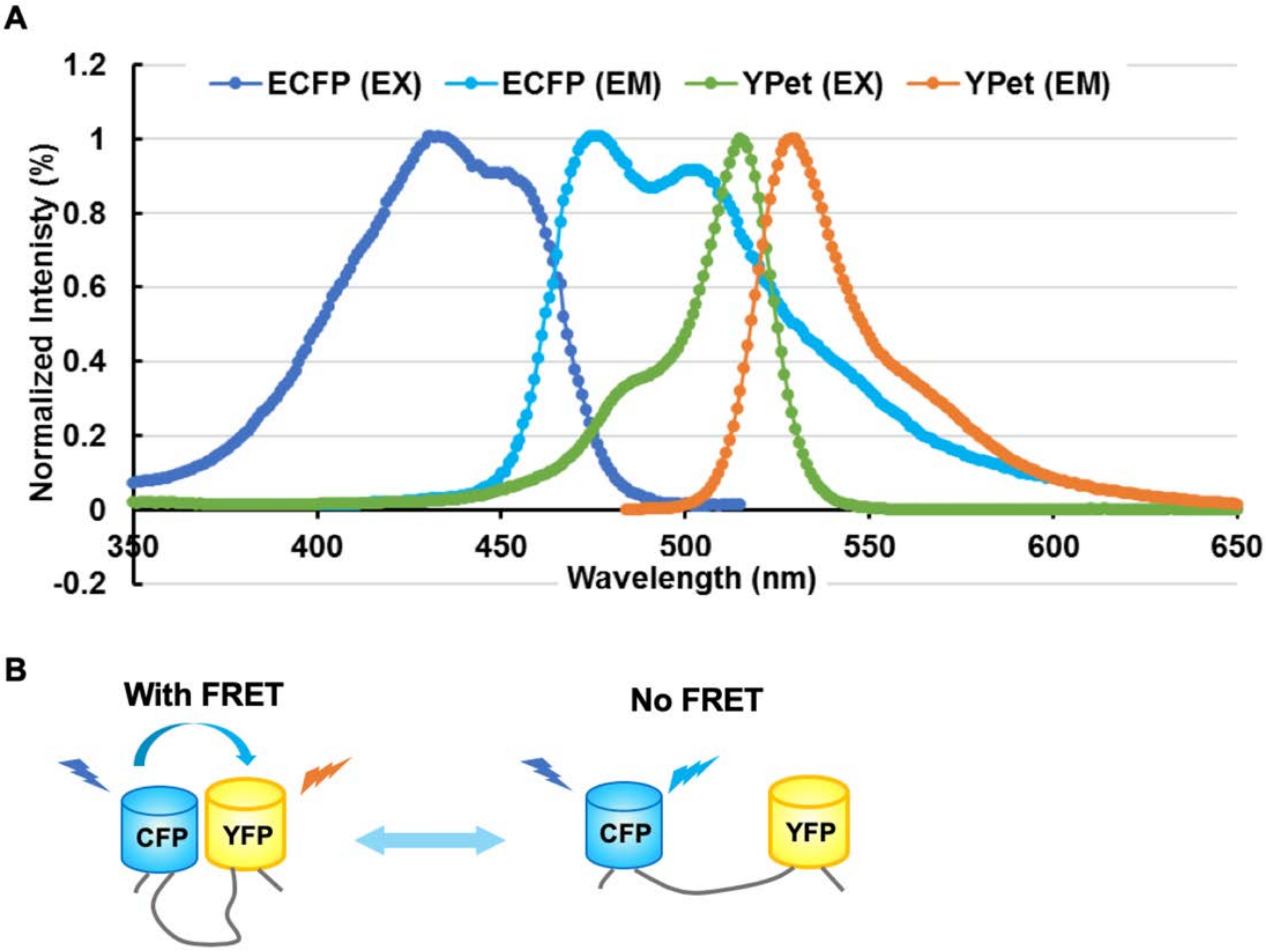

Supplementary Figure S1.

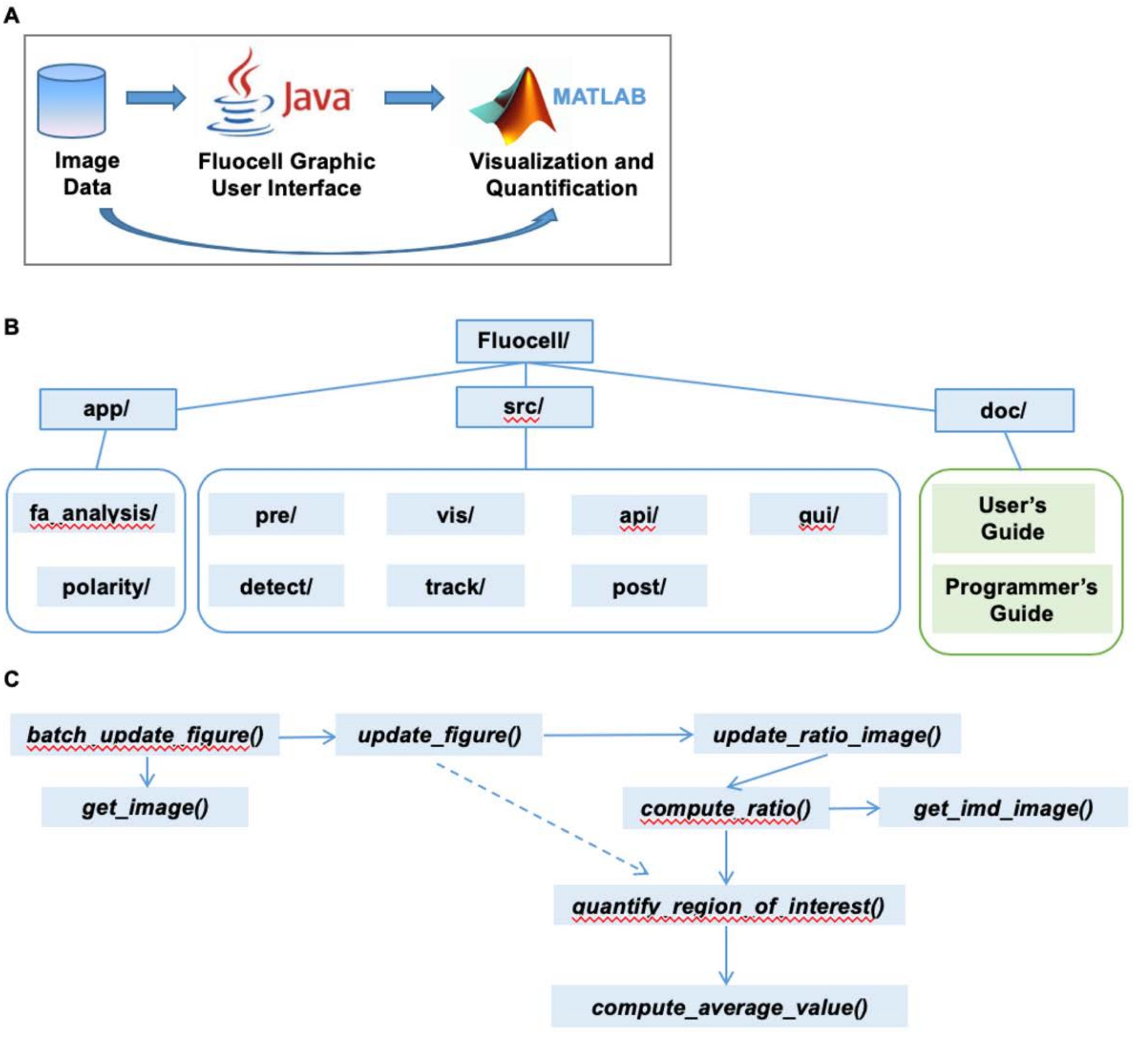

Supplementary Figure S2.

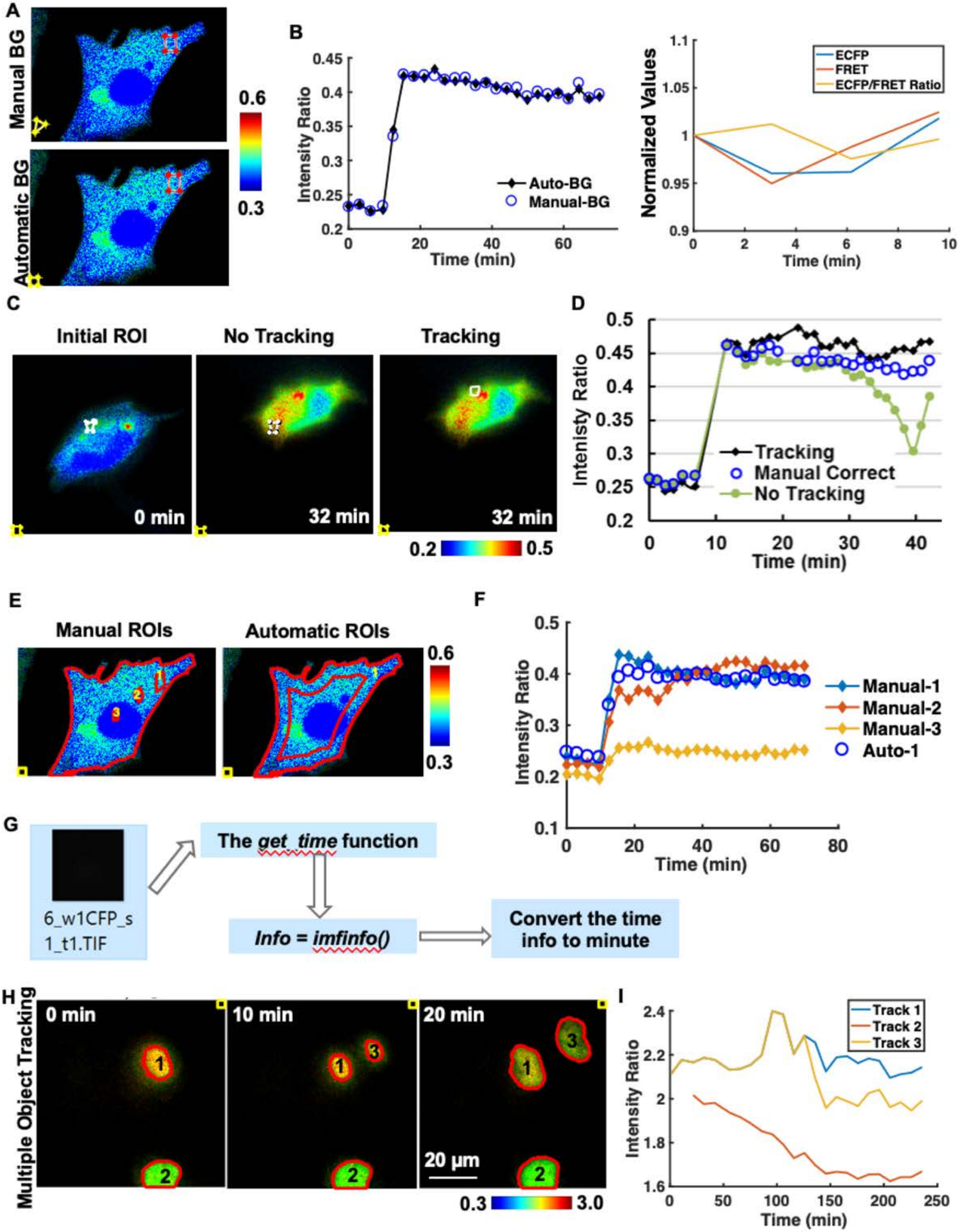
A
Manual BG
Automatic BG
0.6
0.3
B
Intensity Ratio
Auto-BG
Manual-BG
Time (min)
Normalized Values
ECFP
FRET
ECFP/FRET Ratio
Time (min)
C
Initial ROI
No Tracking
Tracking
0 min
32 min
32 min
0.2
0.5
D
Intenisty Ratio
Tracking
Manual Correct
No Tracking
Time (min)
E
Manual ROIs
Automatic ROIs
0.6
0.3
F
Intensity Ratio
Manual-1
Manual-2
Manual-3
Auto-1
Time (min)
G
6_w1CFP_s
1_t1.TIF
The get_time function
Info = imfinfo()
Convert the time
info to minute
H
Multiple Object Tracking
0 min
10 min
20 min
20 μm
0.3
3.0
I
Intensity Ratio
Track 1
Track 2
Track 3
Time (min)

Figure S3.

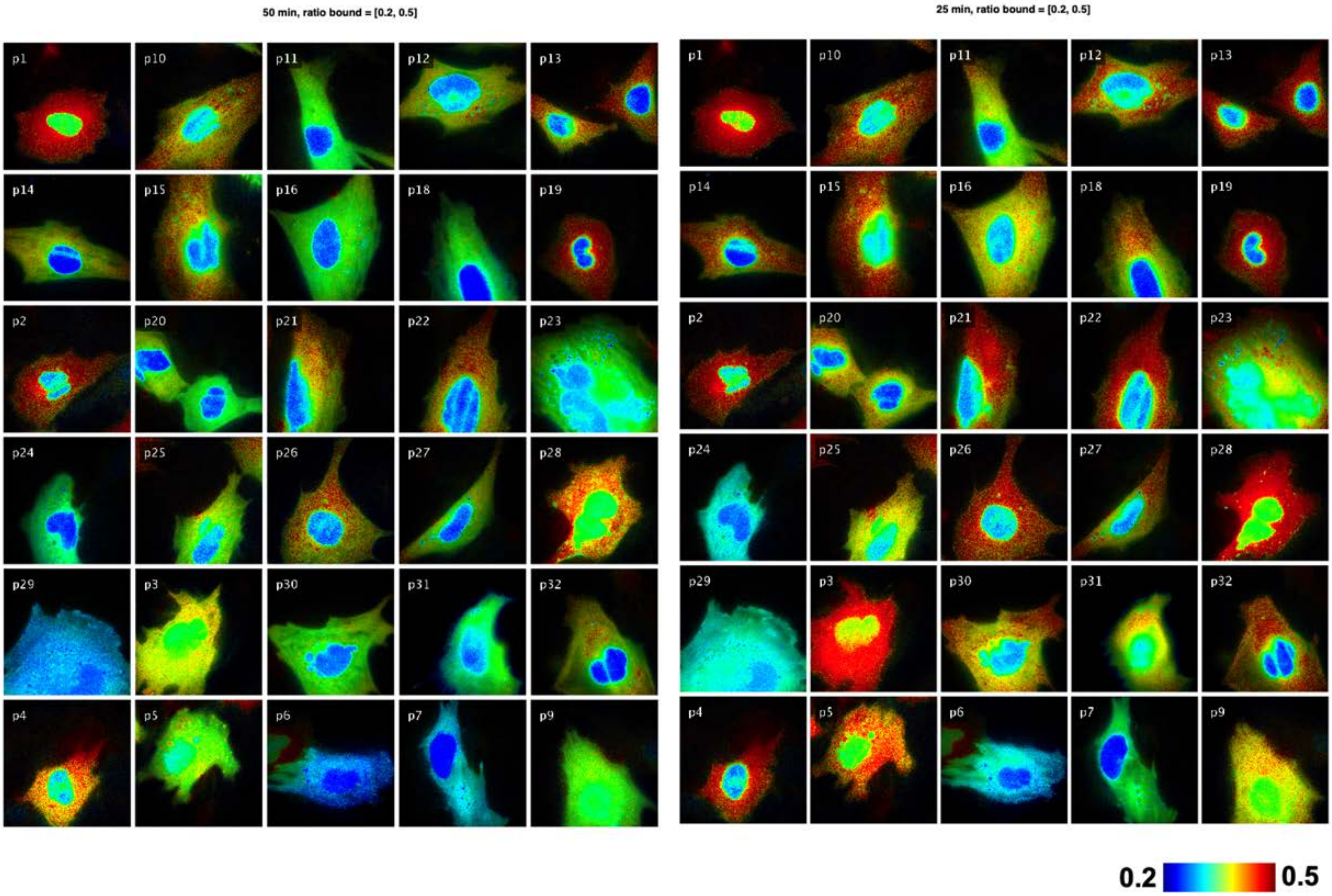

Supplementary Figure S4. Group image view function of Fluocell.

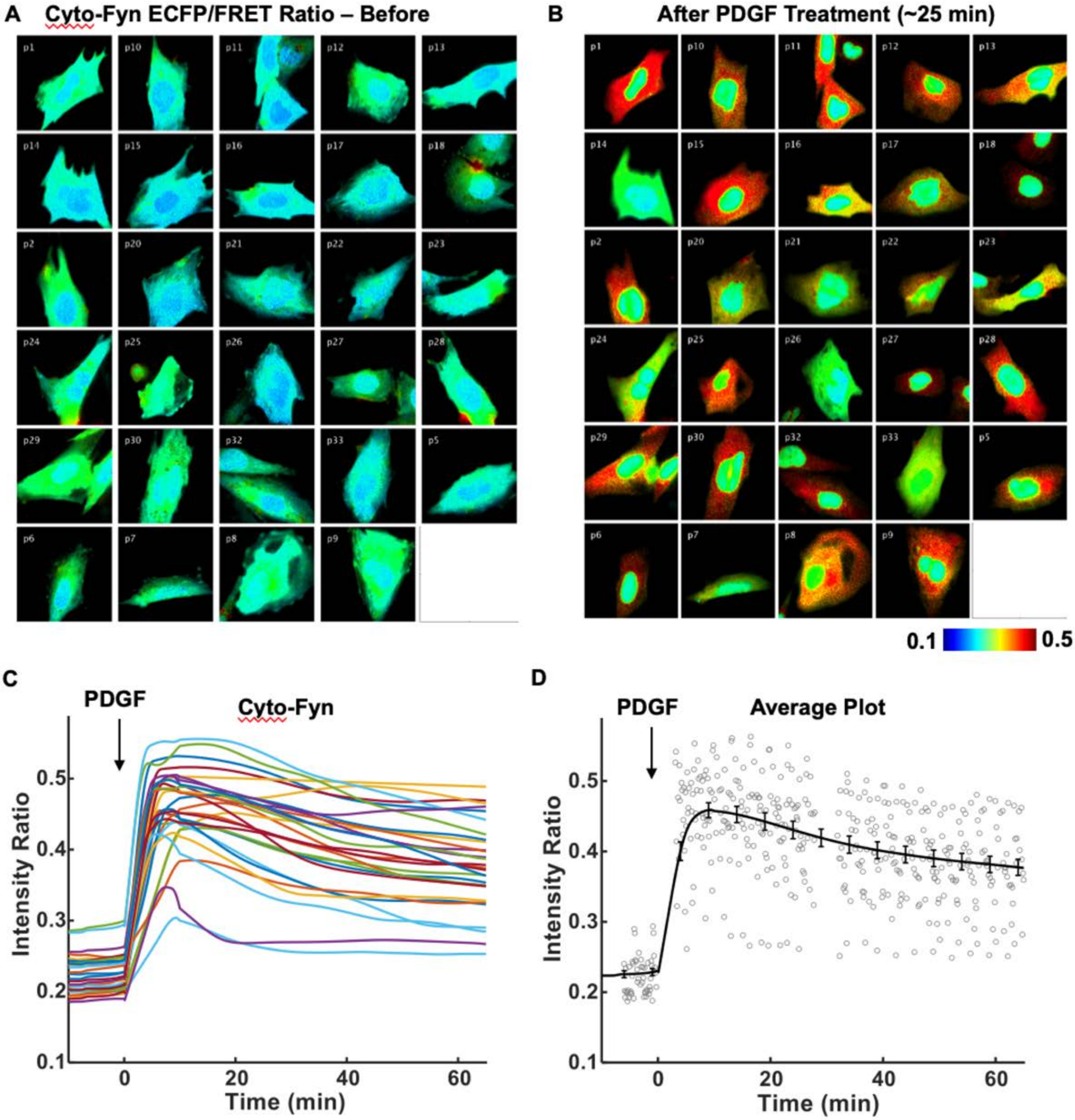

Supplementary Figure S5.

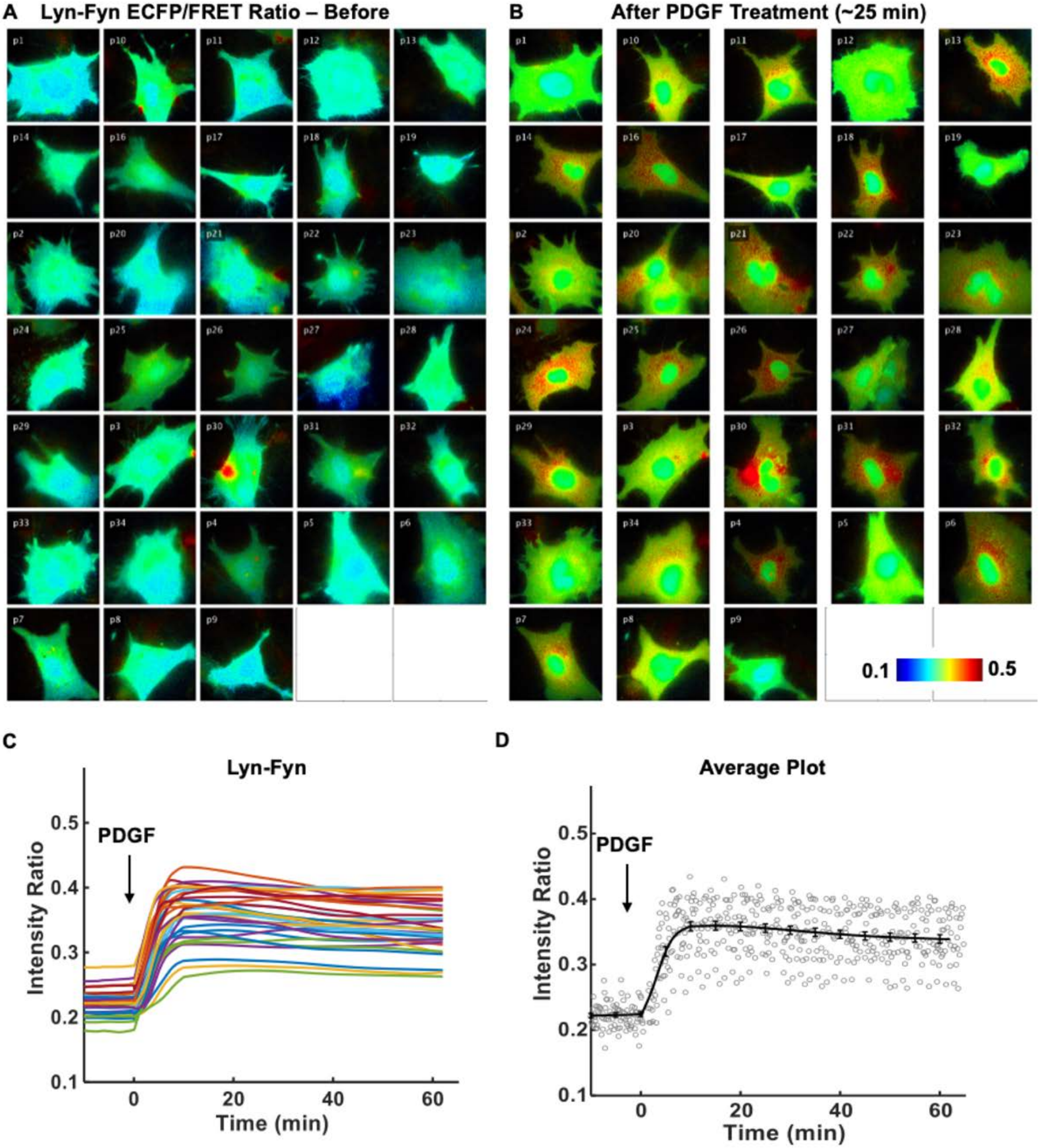

Supplementary Figure S6.

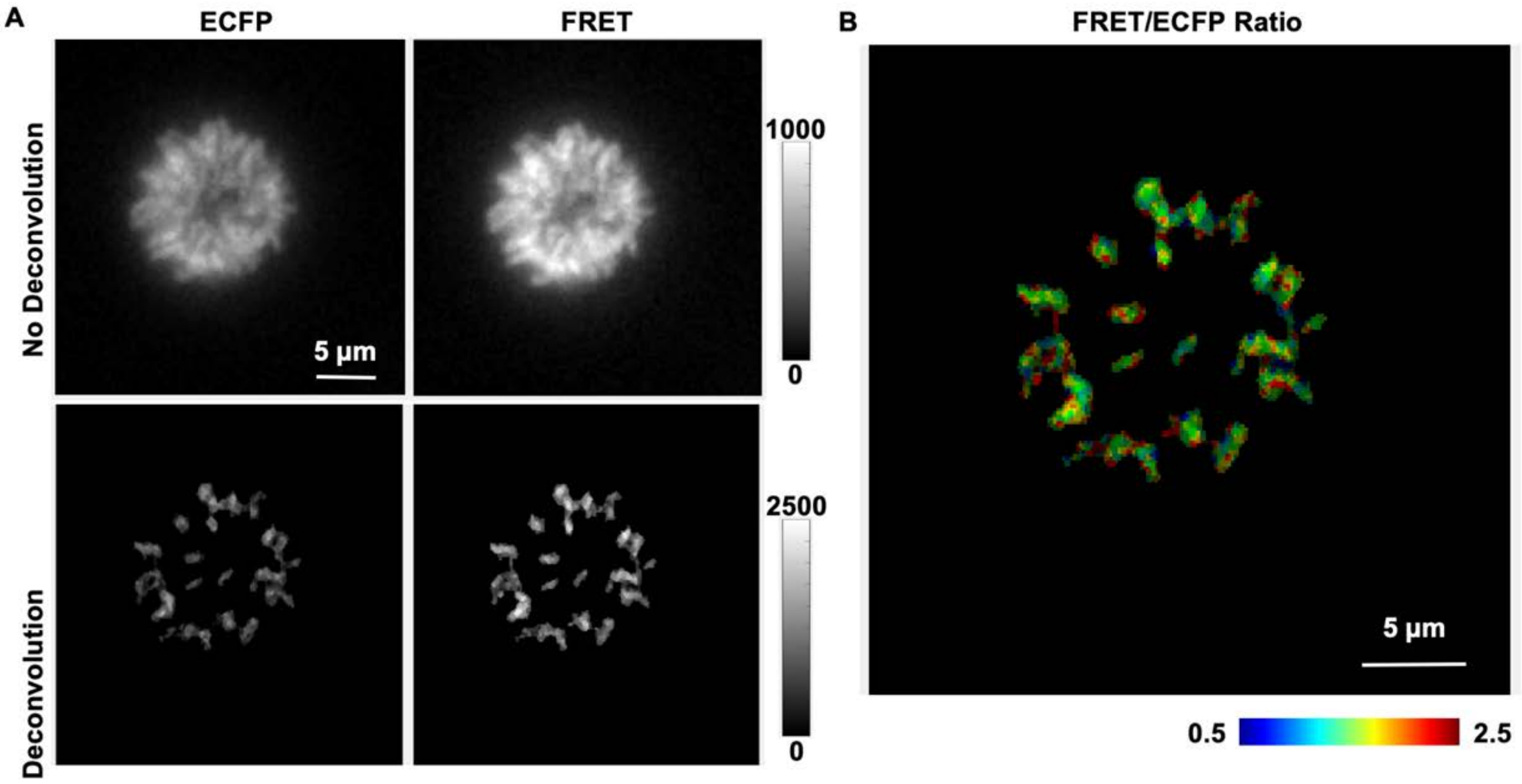

Supplementary Figure S7.
